# Bibliometric and visual analysis of global research trends and frontiers in the tumor microenvironment and immune escape

**DOI:** 10.1097/MD.0000000000049728

**Published:** 2026-07-10

**Authors:** Xinyu Feng, Xiumei Sun, Han Bi, Qifu Bo, Ao Li, Yiyi Ding, Meng Gao, Jinrong Liu, Lingling Wang, Yuying Ren, Xiaoyu Zhao, Wenhao Wang

**Affiliations:** a Department of Comprehensive Oncology, Affiliated Hospital of Shandong Second Medical University, Weifang, China; b School of Clinical Medicine, Shandong Second Medical University, Weifang, China; c Department of Oncology, Heze Municipal Hospital, Heze, China.

**Keywords:** bibliometric analysis, CiteSpace, immune escape, immunotherapy, tumor microenvironment

## Abstract

Research on the tumor microenvironment (TME) and immune evasion profoundly influences tumor progression and therapeutic efficacy. Despite rapid growth in related studies, there is a lack of bibliometric analysis to systematically reveal their knowledge structure, research hotspots, and evolutionary trends. Using CiteSpace, we conducted a multidimensional bibliometric and visual analysis of TME- and IE-related literature retrieved from the Science Citation Index (Web of Science). Among 2727 relevant publications published in 654 academic journals, contributions came from 2974 institutions across 87 countries/regions. The number of publications and citations increased annually, forming an interdisciplinary collaboration network. China (1430 publications, 52.44%) and the United States (693 publications, 25.41%) dominated the field, with the most prolific institution (Fudan University, 133 publications) and author (Wang Wei, 21 publications) both based in China. Close collaborations were observed among countries, institutions, and core authors. Frontiers in Immunology published the most articles (4.4%), while Cancer Research ranked first in co-citations (1894 times), establishing them as the 2 most influential journals in tumor microenvironment and immune escape research. Keyword analysis revealed multifaceted research themes (*Q* = 0.7486), with the top 13 clusters encompassing multiple dimensions, including mechanism research, clinical treatment, and specific tumor type analysis. Hot topics varied across developmental stages. This study is the first to integrate a multidimensional visual analysis of the knowledge landscape in tumor microenvironment and immune escape research. The field has exhibited rapid growth, with diversified research content and domains. Investigations into underlying mechanisms remain enduring, while tumor-specific analyses and immunotherapy research, alongside clinical applications, have emerged as key focal points and challenges. The findings further highlight the translational potential of combined therapeutic strategies targeting TME-immune interactions.

## 1. Introduction

The tumor microenvironment (TME) is a complex and dynamic ecosystem composed of tumor cells, immune cells, stromal cells, extracellular matrix, and signaling molecules.^[1]^ It plays a pivotal role in tumor initiation, progression, metastasis, and treatment. The immune system, as the body guardian, has indispensable functions in confronting tumor cell invasion. Within the TME, tumor cells and immune cells interact closely, engaging in intricate and coordinated cross-talk.^[2–5]^ Recent advances have further highlighted the critical role of tumor-infiltrating lymphocytes in this dynamic interplay, with their phenotypic diversity and functional modulation shaping the immune response within the tumor microenvironment.^[6]^ A variety of intrinsic and extrinsic factors significantly modulate the functionality of immune cells, impairing their ability to accurately recognize and eliminate malignant cells, ultimately leading to “immune escape (immune evasion [IE]).” Among these factors, cytokines produced by diverse immune cell populations play a central role in regulating tumor-stroma interactions and shaping the antitumor immune response.^[7,8]^ Cytokine-producing cells, including various T cell subsets, natural killer cells, and antigen-presenting cells, orchestrate the complex communication network within the TME, influencing both immune activation and suppression.^[8]^

Previous studies have demonstrated that the TME is not merely a passive scaffold for tumor growth but an active regulator involved in the process of immune escape.^[9]^Tumors evade immune surveillance and elimination through various mechanisms, including the upregulation of immune checkpoint molecules (e.g., PD-1 (programmed cell death protein 1)/programmed death-ligand 1(PD-L1), CTLA-4),^[10]^ recruitment of immunosuppressive cells (e.g., regulatory T cells, myeloid-derived suppressor cells [MDSCs]),^[11,12]^ and metabolic reprogramming.^[13]^ Although immunotherapies such as immune checkpoint inhibitors, adoptive cell therapies (e.g., Chimeric Antigen Receptor T-cell [CAR-T]), and cytokine-based treatments have achieved significant clinical success, a considerable proportion of patients still exhibit primary or acquired resistance,^[5]^ which is often mediated by the TME. A deeper understanding of the interaction mechanisms between the TME and immune escape is crucial for advancing cancer immunotherapy.

The tumor microenvironment and immune escape (TME-IE) represent a continuously evolving area of academic focus. Numerous basic and clinical studies related to etiology, pathology, and therapeutics are published annually in various academic journals. A systematic and quantitative assessment of research trends in this field is essential for identifying key advancements, emerging frontiers, and knowledge gaps. Bibliometrics, as an independent discipline, is widely applied in literature analysis.^[14]^ During this process, quantitative evaluations are conducted on authors, affiliated countries, keywords, journals, institutions, references, and other metadata. Such analyses effectively map the developmental trajectory of the research field, track collaborative networks, and reveal evolving trends in scientific focus.^[15,16]^ Furthermore, leveraging modern computational techniques, graphical and visual representations enable the visualization of analytical results. This approach facilitates the identification of underlying relationships among different variables: such as common research themes among authors, institutional research priorities, and novel directions in existing studies: thereby enhancing the comprehensiveness and objectivity of research findings.^[17]^

Although numerous reviews have summarized relevant biological mechanisms, few studies have conducted a comprehensive bibliometric analysis of global publication trends, citation networks, and emerging hotspots in the TME-IE research domain. This study employs bibliometric analytical tools to systematically evaluate existing research outputs, identifying key contributors (countries, institutions, authors, and journals) that have driven advancements in this field. By mapping the knowledge structure and temporal evolution patterns, our findings will assist researchers, clinicians, and policymakers in recognizing high-impact research directions, fostering interdisciplinary collaboration, and providing a strategic roadmap for future investigations.

## 2. Materials and methods

### 2.1. Data acquisition

As a high-quality digital literature database, the Science and Technology Citation Index (Web of Science [WOS]) can index research datasets from a large number of journals.^[18]^ Therefore, WOS is considered to be the most suitable database for bibliometric analysis and has been accepted by many researchers.^[19]^ We searched the core collection (WOS Core Collection) of the WOS database on September 21, 2024, and the search format was determined as follows: TS = (“immune escape” or “immune evasion” or “immune evasive” or “antigenic escape” or “immunological escape”) and (tumor or cancer) and (microenvironment* or micro-environment*), the document type was limited to the article, the language was English, and the publication date was set to all years. After data examination and de-duplication, a total of 2727 articles were included in the study (Table S1, S2, S3, Supplemental Digital Content 1). The key data of each study (title, year of publication, author, nationality, institutional affiliation, journal, keywords, abstracts, etc) were imported into Microsoft Excel for further analysis. The above searches were completed within one day to exclude any impact of database updates on the results of the study.

### 2.2. Research methods

As a Java application for bibliometric analysis, CiteSpace focuses on the dynamic visualization of bibliometrics.^[20,21]^ CiteSpace obtains the time map and timeline view by applying the similarity algorithm to time slicing, so that it can clearly outline the process of knowledge evolution and the historical span of documents in a certain cluster, and understand the development process and trends in this field. In this study, CiteSpace (version 6.2. R6) as the main tool, and the organization, journals, authors, co-cited authors, references and keywords related to the field of cancer immune escape were visually analyzed.

This study adopts a bibliometric research approach, with data sourced from the publicly available academic database WOS. By analyzing published literature data, it focuses on macro-level trends in disciplinary development. It does not involve personal privacy, sensitive information, or negative evaluations of individual authors or institutions, nor does it risk privacy breaches. As such, this study does not require ethical review and does not involve participant recruitment or informed consent. All figures and tables presented in this study are generated from standard bibliometric analyses and are intended solely for scientific mapping of the research field. They are not intended as, nor should they be construed as, promotion or endorsement of any specific journals, institutions, countries, or authors.

## 3. Results

### 3.1. Annual publications and citations

**Figure 1.**
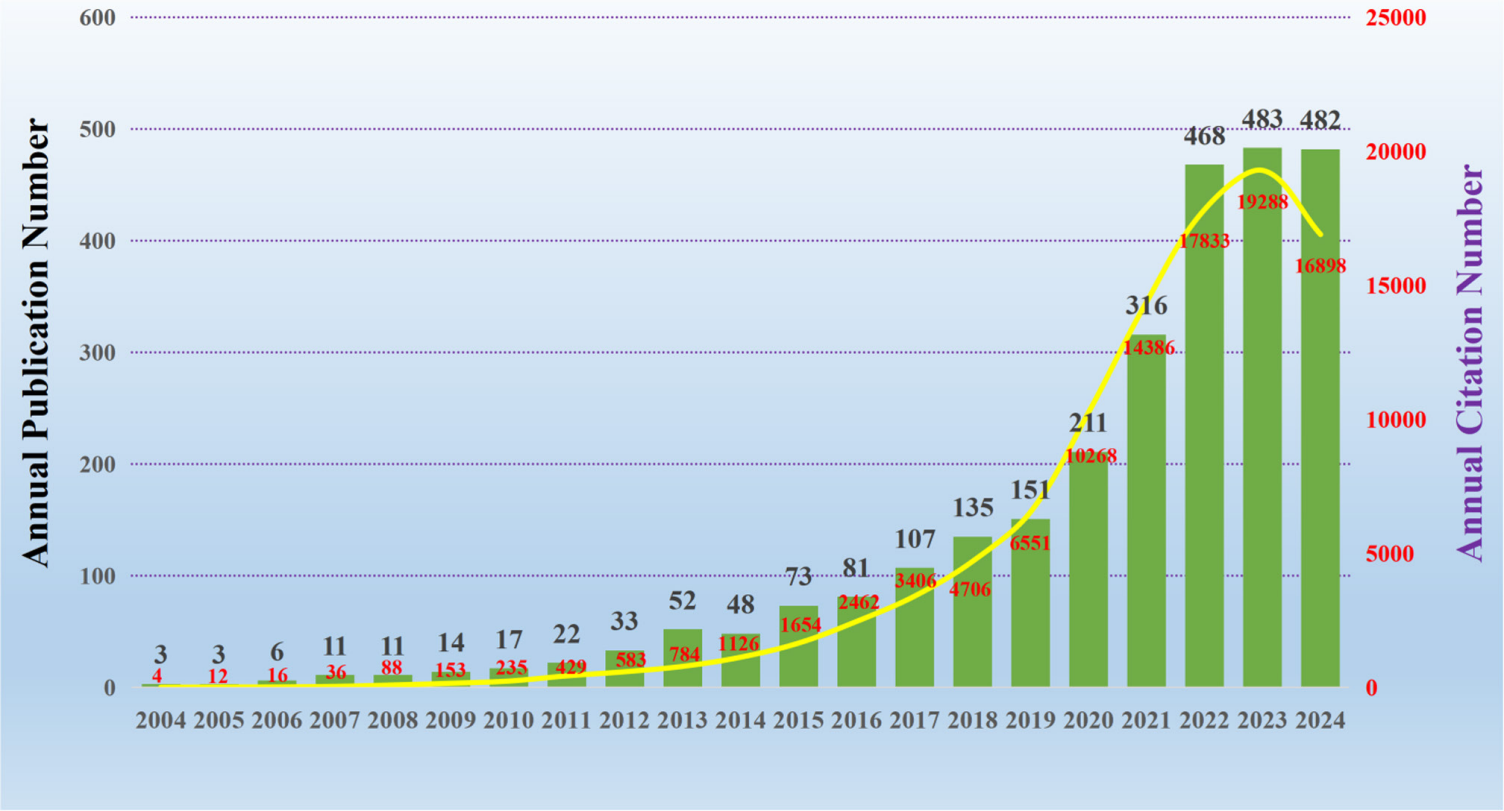
The annual number of publications and citations on TME-IE between 2004 and 2024. TME-IE. = tumor microenvironment and immune escape.

The dynamics of the number of publications can reveal the research progress and academic attention in the field of TME-IE. over a specific period of time. In October 2024, we searched and downloaded a total of 2727 TME-IE-related publications. In terms of data years, the first study to discuss TME-IE directly appeared in 2004. Since then, scholars have carried out continuous research on tumor immune escape. The number of articles published has continued to grow since 2004. Specifically, from 2001 to 2010, the number of related research papers increased slowly, indicating that TME-IE research was in the early stage. From 2011 to 2016, the number of research papers in this domain increased, indicating that the field has begun to attract academic attention. From 2017 to 2024, the number of research papers in this field increased rapidly; the highest annual output reached 483, the overall average annual growth rate was the highest, and TME-IE has become a popular research field. (Fig. 1)

In terms of the number of citations, the number of citations showed a rapid upward trend, maintained sustained and steady growth from 2004 to 2018, and reached 6551 in 2018. Since 2019, the annual number of citations has exceeded 10,000. In particular, in 2023, the number reached a peak of 19,288, with an average of approximately 40 citations per publication. (Fig. 1)

### 3.2. Analysis of journals and co-cited journals

A total of 654 academic journals have published articles about TME-IE. As shown in Table 1, the top 10 journals accounted for nearly 1/4 of the total publications, of which “Frontiers In Immunology” (*IF* = 5.7) published the largest number of articles (120,4.4%), followed by “Cancer Research,” “Frontiers In Oncology,” and “Journal for Immunotherapy of Cancer.” Compared with other journals, the top 4 journals contributed more than 70 articles, accounting for more than 2.5%, indicating that they have a special status in the TME-IE field. Among the 10 journals with the most published literature, the average impact factor of these 10 journals is 7.532, and the impact factor of 3 journals is more than 10.0. Research on TME-IE is becoming increasingly important in academia because of its key role in cancer treatment and in-depth understanding of the mechanism of immune escape. With increasing research, the influence of and innovation in the field of TME-IE is gradually becoming the core of oncology research, promoting the development and innovation of cancer treatment strategies.

**Table 1:** Ranking of the top 10 journals and co-cited journals involved in the TME-IE domain.

Publication titles	Record count	% of 2,727	IF	Cited journal	Record counts
Frontiers in Immunology	120	4.4	5.7	Cancer Research	1894
Cancer Research	84	3.08	12.5	Nature	1721
Frontiers in Oncology	72	2.64	3.5	Clinical Cancer Research	1606
Journal for Immunotherapy of Cancer	71	2.604	10.3	Cell	1524
Cancers	61	2.237	4.5	PNAS	1500
Oncoimmunology	55	2.017	6.5	Nature Medicine	1332
Cancer Immunology Immunotherapy	50	1.834	4.72	Science	1311
Cancer Letters	46	1.687	9.1	Nature Reviews Cancer	1276
Nature Communications	39	1.43	14.7	Nature Communications	1203
Scientific Reports	39	1.43	3.8	Cancer Cell	1199

**Figure 2.**
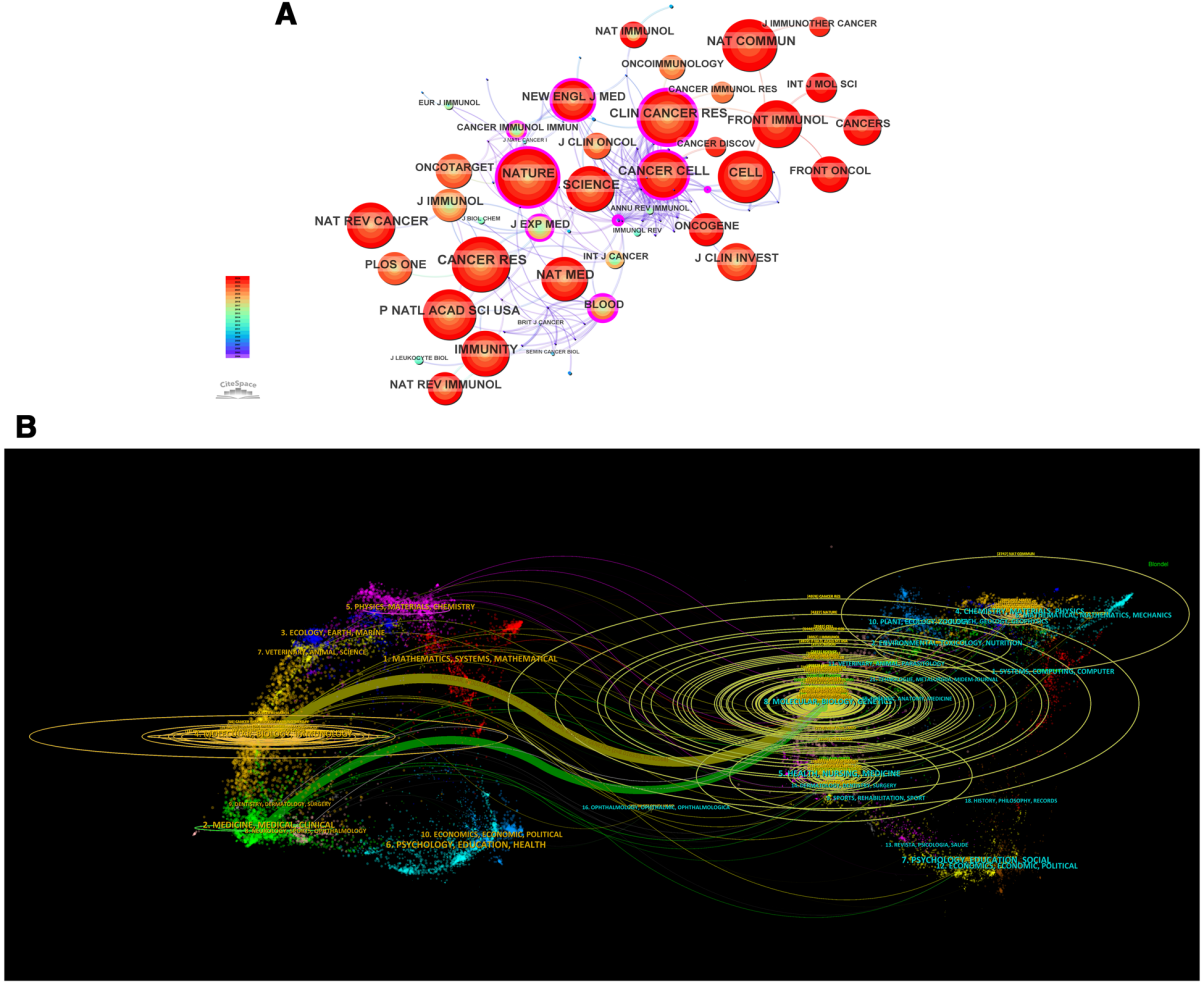
(A) The map of co-cited journals; (B) A dual-map overlay of journals that published literature in the TME-IE field. TME-IE. = tumor microenvironment and immune escape.

In the list of cited journals, “Cancer Research” ranks first with 1894 co-citations, whereas “Nature” and “Clinical Cancer Research” are cited 1721 times and 1606 times, respectively. These high citation numbers, to some extent, show that these journals are academically competitive and are considered mainstream journals in the TME-IE field. The network diagram of cited journals drawn through CiteSpace contains 107 nodes and 286 links. Figure 2A shows that journal publications have a wide range of concerns, including cancer biology, immunology, molecular biology and genetics, clinical medicine and transformation research, and several comprehensive interdisciplinary studies. Because the cited journals provide a theoretical basis for the cited journals, these diversified tracks show that the discipline center of the journals has shifted from a single discipline to a multidisciplinary cluster.

Fig. 2B shows the double-graph superposition of journals published in the field of TME-IE. This method uses 2 diagrams at the same time. On the left is the category of the cited journal, and on the right is the discipline of the cited journal. The reference link displays the flow of the reference dataset. Because the cited journals put forward their views on the cited journals, the diagram reflects the citation relationships between disciplines to a certain extent. Generally, there are 2 main citation paths. The articles published in the light orange path “Molecular, Biology, Immunology” and the grass green path “Medicine, Medical, Clinical” and “Neurology, exercise, Ophthalmology” tend to cite journals in the field of “Molecular, Biology, Genetics.”

### 3.3. Cooperative relationship network

#### 3.3.1. Country and institutional cooperation network

A total of 87 countries participate in research in the field of TME-IE. Details of the top 10 countries and institutions are shown in Table 2. China and the United States dominate, with 1430 and 693 publications, respectively. This phenomenon may be closely associated with the increased investment in scientific research within the public health domain by China and the United States in recent years, which has facilitated advancements in research equipment development and the cultivation of scientific personnel. Germany is the third most prolific country, with 198 articles published, followed by Italy (131) and Japan (105). The top 6 countries all sent more than 100 articles, indicating that they have made a significant contribution to the research results.

**Table 2:** Ranking of the top 10 countries and institutions involved in the TME-IE domain.

Country	Publications	Centrality	Institution	Public-ations	Cent-rality
Peoples R China	1430	0.09	Fudan University	133	0.02
USA	693	0.18	Sun Yat-Sen University	110	0.02
Germany	198	0.09	Chinese Academy of Sciences	106	0.15
Italy	131	0.09	Harvard University	99	0.02
Japan	105	0.02	Shanghai Jiao Tong University	99	0.02
England	101	0.29	University of TEXAS System	89	0.26
France	89	0.75	Southern Medical University China	73	0.01
South Korea	79	0	Chinese Academy of Medical Sciences Peking Union Medical College	69	0.01
Canada	66	1.01	Harvard Medical School	68	0.08
Switzerland	60	0.33	Utmd Anderson Cancer Center	68	0.04

**Figure 3.**
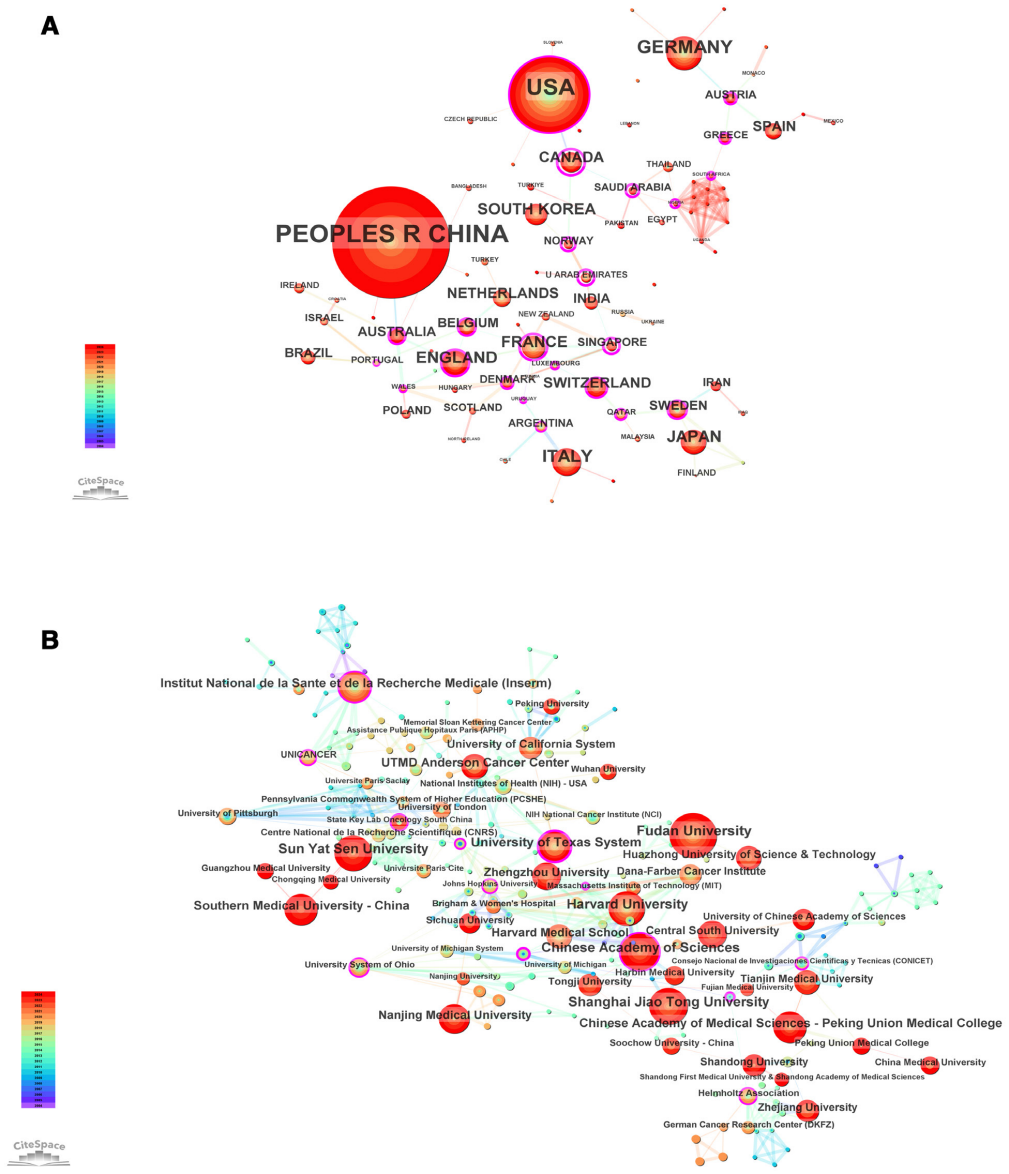
Network diagram of national cooperation (A) and institutional cooperation (B) in the field of TME-IE. TME-IE. = tumor microenvironment and immune escape.

A total of 2947 institutions are involved in this field. As shown in Table 2, the top 3 institutions with the largest number of published TME-IE papers are FUDAN UNIVERSITY (133,110 and the CHINESE ACADEMY OF SCIENCES. CiteSpace was used to generate an agency collaboration network diagram (Fig. 3B). The cooperation between the institutions with the largest number of forms is very close. Among them, UNIVERSITY OF TEXAS SYSTEM, which ranks sixth in the number of published papers, cooperates most closely with other institutions, followed by CHINESE ACADEMY OF SCIENCES (0.15) and HARVARD MEDICAL SCHOOL (0.08).

#### 3.3.2. Author cooperation network

**Figure 4.**
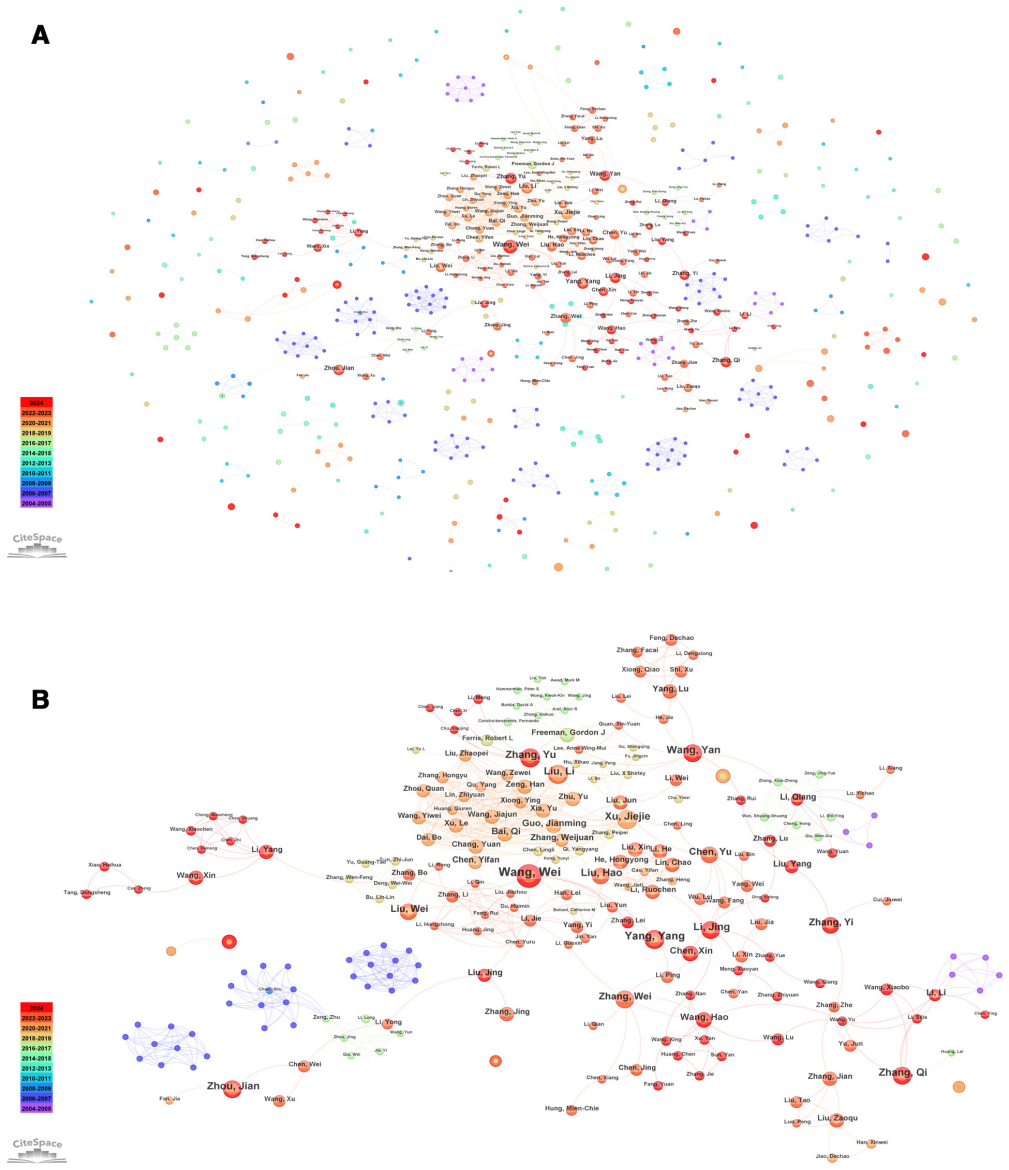
Author collaboration (A) and core collaboration (B) network map.

The author collaboration network enables us to find the core authors in this field and determine the intensity of their cooperation. The author collaboration network shown in Figure 4A has 579 nodes and 1204 links with a network density of 0.0072. The size of the nodes represents the number of papers published by the authors, and the links between the nodes represent the connections or cooperation between the authors. For the observability of the network graph, the threshold is set to 2, that is, only the authors with more than 2 posts are displayed in the graph (Fig. 4B shows the core cooperative network). The atlas shows that, with the exception the relatively large cooperative network formed by the high-yield authors in the network center, the other authors publish articles in the form of individual articles or small group cooperation.

Table 3 lists the information on the top 10 authors. Wang and Wei are the most prolific authors, with 21 articles published, followed by Xu, Jiejie and Yang, Yang, with 13 articles all published. The total number of other authors published was more than 10. In terms of the total number of citations, the rankings were Liu, Hao, Xu, Jiejie, Yang, Yang and Liu, Li. Taking into account the average number of citations per paper, the top 3 authors are Liu, Hao (58.38), Xu, Jiejie (46.73) and Liu, Li (40.08). The centrality of the node indicates the degree of influence in the network. The thicker the purple aperture is, the greater the centrality. The centrality of Wang, Wei, Liu and Hao is the highest in this research field, indicating that the nodes of Wang, Wei, Liu and Hao are key nodes in the coauthor analysis diagram.

**Table 3:** List of the top 10 authors with published articles.

Author	Total publication	Number of citations	Average citation per paper	Centrality
Wang, Wei	21	408	19.43	0.07
Xu, Jiejie	15	701	46.73	0.03
Yang, Yang	15	594	39.60	0.04
Liu, Hao	13	759	58.38	0.07
Liu, Li	13	521	40.08	0.00
Zhang, Yu	12	392	32.67	0.01
Zhang, Qi	12	385	32.08	0.00
Li, Jing	12	328	27.33	0.04
Wang, Yan	10	273	27.30	0.05
Zhou, Jian	10	215	21.50	0.00

The research team led by Professor Wei Wang has developed a novel chemo-immunotherapeutic system for antitumor therapy, which integrates a tetrazine-modified anti-PD-L1 antibody with a tetrazine-activatable prodrug (αPD-L1TZ plus NP@PDOX). This innovative approach enables site-selective bioorthogonal activation of the prodrug, disrupts PD-L1/PD-1 interactions, and induces immunogenic cell death. The mutually reinforcing feedback between PD-L1 blockade and prodrug activation significantly amplifies the antitumor efficacy of this nanoplatform.^[22]^ These investigations focus on developing novel immunotherapeutic strategies and enhancing the immune system antitumor capacity through tumor microenvironment modulation, thereby overcoming tumor IE. Such research directions not only demonstrate remarkable innovation but also hold substantial potential to shape the future landscape of cancer therapeutics.

This study also constructed collaborative networks among relevant countries/regions, institutions, and authors. From a network perspective, centrality metrics are of paramount importance. Centrality represents the strength of connections between a given node and others within the entire network; high centrality indicates that a key node exerts substantial influence over network relationships. As illustrated in Figures 3A and 3B, dominant entities in the TME-IE field, including nations (China, the United States) and institutions (University of Texas System, Chinese Academy of Sciences, Harvard Medical School): demonstrate robust collaborative ties. Figure 4B further reveals that highly productive authors at the network core form relatively large collaborative clusters, while others predominantly publish either independently or within small research groups. According to global cancer statistics reports ^[23]^, the worldwide distribution of malignancies is influenced by geographic disparities, healthcare resources and capabilities, Human Development Index, and other multifactorial determinants. Consequently, enhancing international and inter-institutional cooperation, improving information sharing among researchers, and fostering a multi-centric developmental paradigm would significantly advance the exploration of the TME-IE domain.

### 3.4. Analysis of subject categories

**Figure 5.**
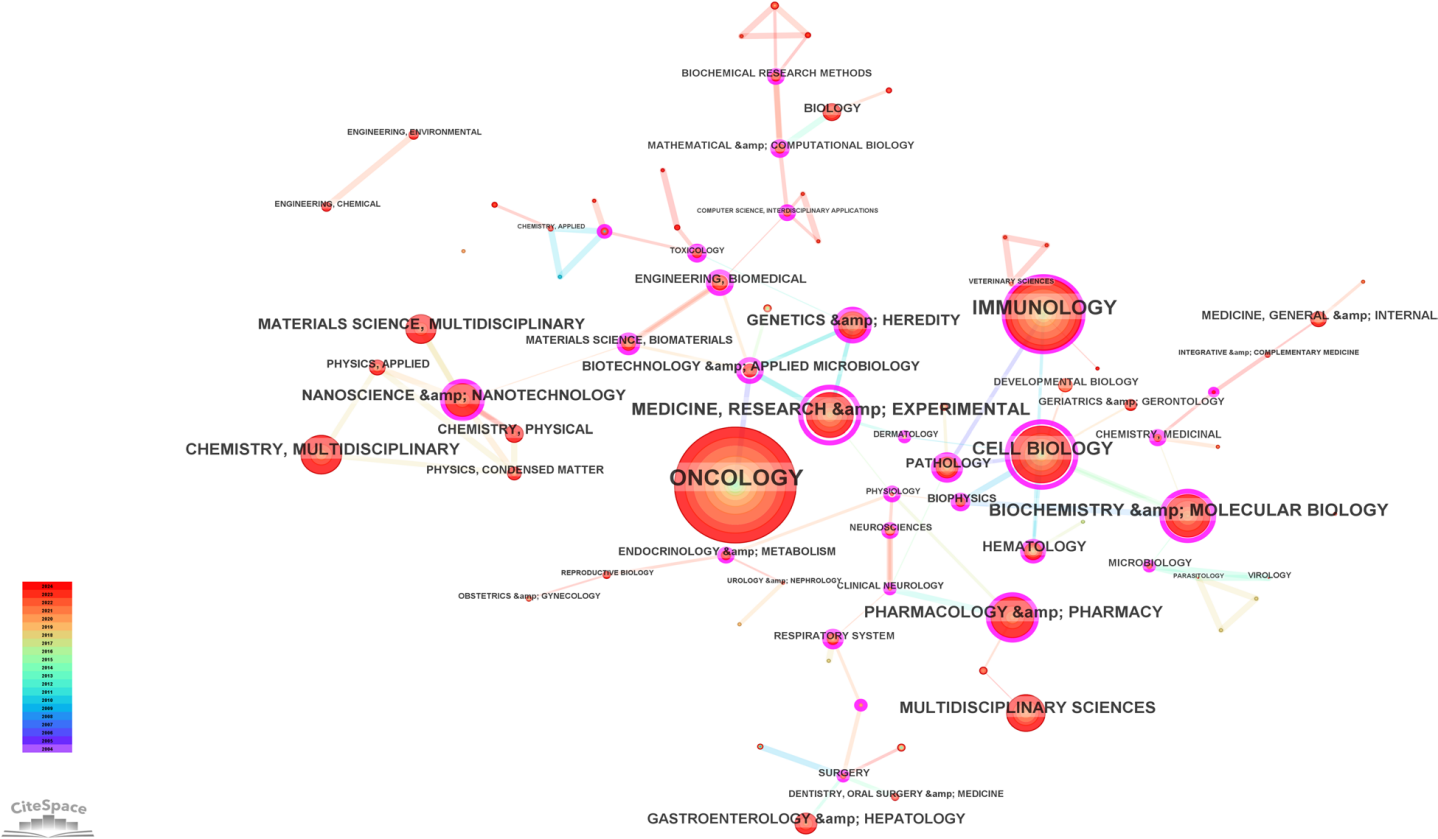
Knowledge map of the category co-occurrence network.

The subject category co-occurrence network knowledge graph visualizes the topic categories that appear at least 5 times (Fig. 5). The occurrence frequency of oncology was the highest (1102 times), followed by immunology (532 times), cell biology (333,240 times), medical research and experiments (240 times) and biochemistry and molecular biology (229 times)(see Table 4). This shows that these subject categories have been the subject of extensive research on TME-IE and are the main contributors in this field. Medical research and experiments ranked highest in terms of intermediary centrality (0.86), followed by biotechnology and applied microbiology (0.71), physiology (0.65), cell biology (0.60) and biochemistry and molecular biology (0.46) (see Table 4). Their bridging role in interdisciplinary research is highlighted. By adjusting the duration of the outbreak to 1 year, 6 categories of outbreak intensity were identified (Table 5), and the emergence of these thematic categories began in 2007. According to the time of the outbreak, developmental biology represents the frontier research department in the field of TME-IE.

**Table 4:** The top 10 subject categories in terms of frequency and betweenness centrality.

Subject categories	Frequency	Subject categories	Centrality
Oncology	1102	Medicine, Research & Experimental	0.86
Immunology	532	BIotechnology & Applied Microbiology	0.71
Cell BiOLOGY	333	Physiology	0.65
Medicine, Research & Experimental	240	Cell Biology	0.6
Biochemistry & Molecular Biology	229	Biochemistry & Molecular Biology	0.46
Multidisciplinary Sciences	174	Engineering, Biomedical	0.41
Pharmacology & Pharmacy	169	Clinical Neurology	0.38
Chemistry, Multidisciplinary	141	Computer Science, Interdisciplinary Applications	0.38
Genetics & Heredity	116	Dermatology	0.36
NAnoscience & Nanotechnology	115	Genetics & Heredity	0.34

**Table 5:** Top 6 subject categories with the strongest citation bursts.

Subject Categories	Yr	Strength	Begin	End	2004–2024
Hematology	2007	4.79	2007	2020	▂▂▂▃▃▃▃▃▃▃▃▃▃▃▃▃▃▂▂▂▂
Immunology	2004	5.34	2008	2009	▂▂▂▂▃▃▂▂▂▂▂▂▂▂▂▂▂▂▂▂▂
Dermatology	2010	3.6	2010	2019	▂▂▂▂▂▂▃▃▃▃▃▃▃▃▃▃▂▂▂▂▂
Oncology	2004	4.14	2011	2012	▂▂▂▂▂▂▂▃▃▂▂▂▂▂▂▂▂▂▂▂▂
Cell Biology	2005	10.3	2016	2017	▂▂▂▂▂▂▂▂▂▂▂▂▃▃▂▂▂▂▂▂▂
Developmental Biology	2021	6.07	2021	2022	▂▂▂▂▂▂▂▂▂▂▂▂▂▂▂▂▂▃▃▂▂

### 3.5. Reference co-citation analysis

**Figure 6.**
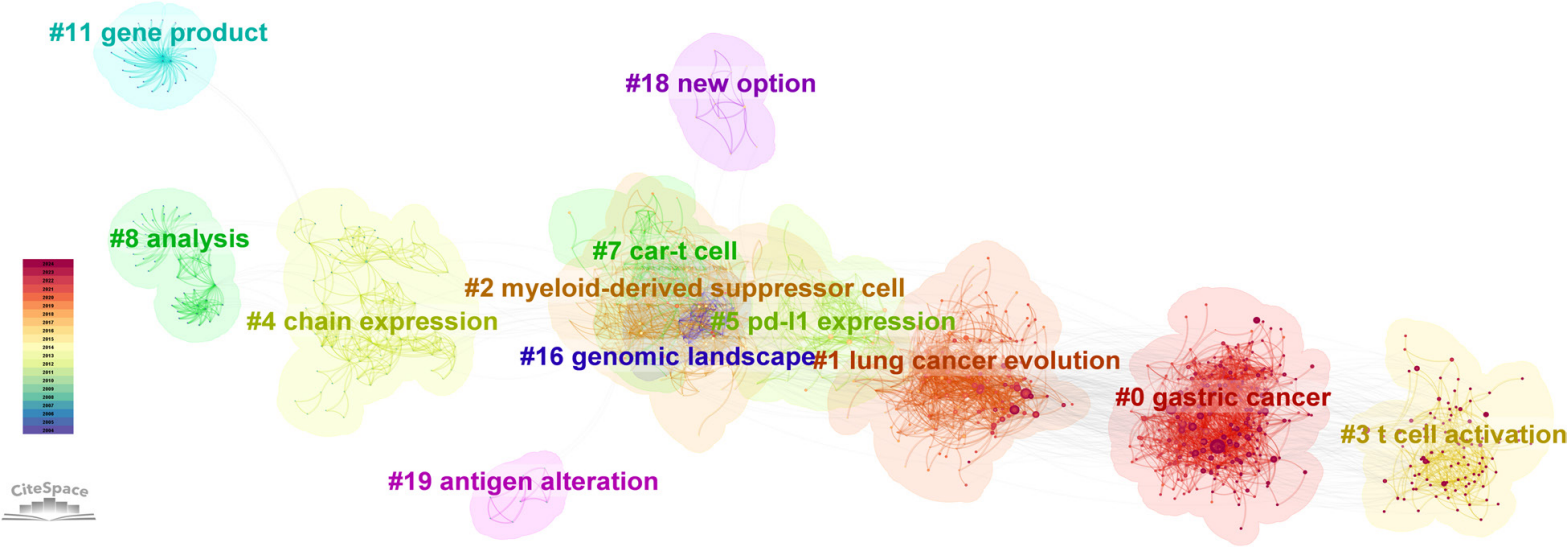
Mapping of the reference co-citation cluster.

Fig. 6 shows the results of the co-citation analysis of 1439 references. In the literature clustering analysis, the topic title of the paper is taken as the clustering data source, and the logarithmic likelihood rate algorithm is used for clustering analysis. Through cluster analysis, 76 clusters were obtained (the top 12 clusters are listed in the chart: gastric cancer, lung cancer evolution, myeloid-derived suppressor cell, T cell activation, chain expression, PD-L1 expression, CAR-T cell analysis, gene product, genomic landscape, new options, and antigen alteration). Modulus *Q* = 0.7351, weighted average silhouette *S* = 0.9073. The subgroups are clearly divided. The co-citation analysis of references reflects the frequently cited papers in academic research in this subject field, and provides basic knowledge support for new research. The smaller the number of clustering tags is, the more important the number of documents contained in the cluster.

Co-citation cluster analysis reveals that while the various clusters appear independent, they are intrinsically interconnected, collectively forming a complex network within the TME-IE research domain.

The research team from Sun Yat-Sen University discovered that the IKZF4/NONO-RAB11FIP3 axis promotes immune escape in gastric cancer by facilitating PD-L1 endosomal recycling, providing a potential therapeutic target for gastric cancer patients.^[24]^ Understanding the evolutionary dynamics of lung cancer and its intricate relationship with the immune system is critical for predicting immune escape pathways and developing effective immunotherapies. Studies indicate that untreated early-stage non-small cell lung cancer exhibits multiple independent IE mechanisms, underscoring the role of the immune system in tumor evolution.^[25]^ MDSCs have garnered increasing attention for their immunosuppressive role in the TME. They promote tumor progression and induce immune escape through multiple mechanisms, making the study of MDSC surface markers and their biological significance potentially valuable for cancer therapy.^[26]^ T-cell activation is pivotal for antitumor immune responses. Investigating its mechanisms can aid in developing immune-boosting strategies. For instance, PD-L1 on dendritic cells plays a crucial role in T-cell activation and tumor immunotherapy.^[27]^ Gene expression patterns may influence tumor immune interactions by modulating surface molecules on tumor cells, thereby affecting immune escape. PD-L1 expression levels strongly correlate with IE capacity,^[28]^ and elucidating its regulatory mechanisms is essential for developing immune checkpoint inhibitors. CAR-T cell therapy, which employs engineered T cells to specifically target and eliminate tumor cells, holds significant promise. Research into its application and optimization for solid tumors is critical for improving therapeutic outcomes.^[29]^ Deep profiling of immune cells and molecular components within the TME helps uncover immune escape mechanisms and provides a scientific foundation for novel treatment strategies. The role of gene products in IE is a major research focus. For example, large-scale genetic knockout screens have identified 182 immune escape-related genes in cancer cells, which may guide future immunotherapies.^[30]^ Genomic studies enhance our understanding of tumor heterogeneity and evolution, which is vital for predicting and overcoming immune escape.^[31]^Developing novel therapeutic strategies, such as drugs targeting specific IE mechanisms, could expand treatment options for cancer patients. Tumor cells can evade immune surveillance by altering antigen expression. Investigating these mechanisms is crucial for devising next-generation immunotherapies.

### 3.6. Keyword analysis: research trends and hotspots

#### 3.6.1. Keyword co-occurrence analysis

**Figure 7.**
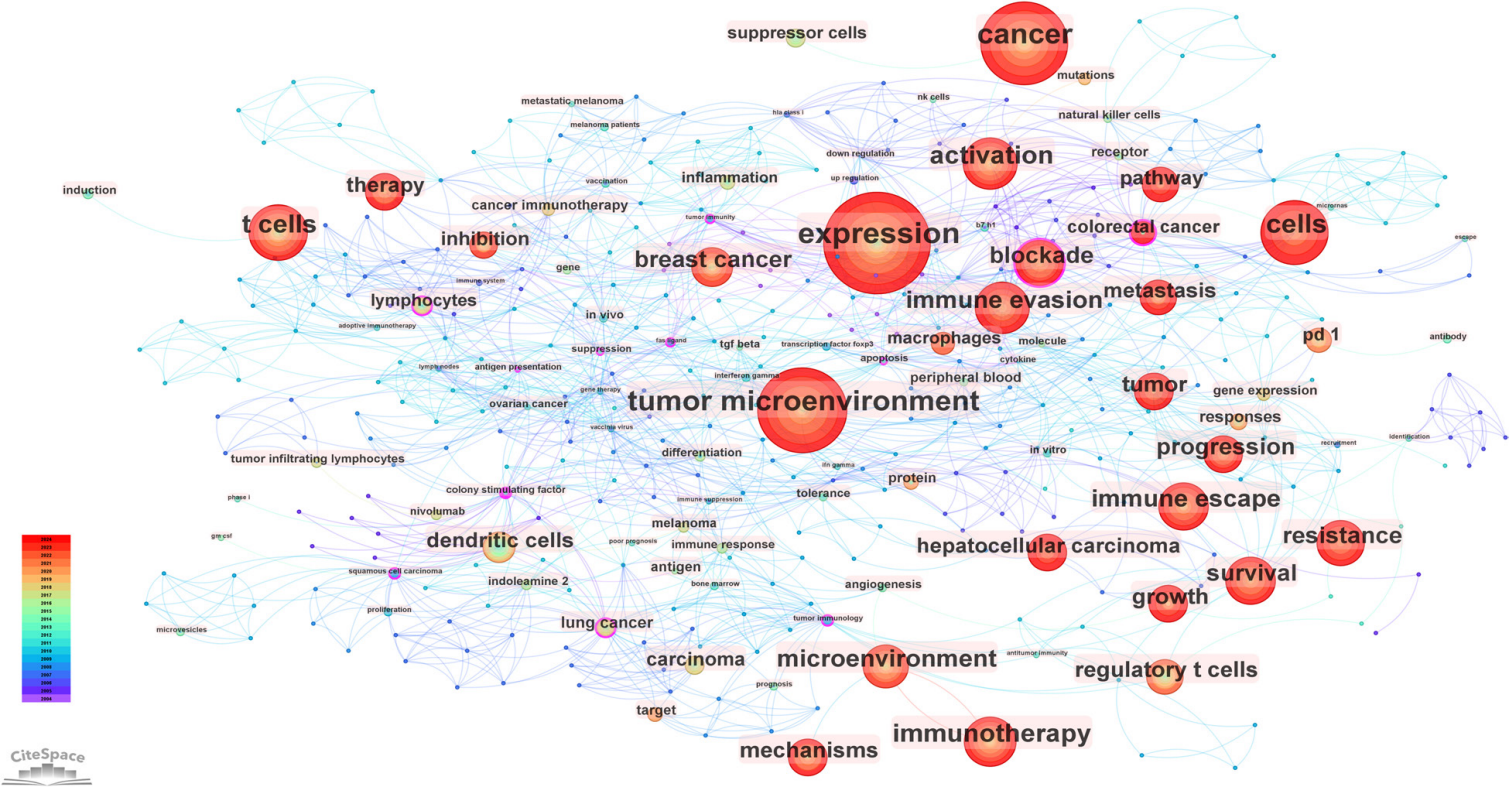
Knowledge mapping of the keyword co-occurrence network in TME-IE research.

Keywords are the basic refinement and core content of the article, whereas keyword co-occurrence analysis is used to identify the main research areas related to TME-IE research. Co-occurrence means that 2 keywords are used together in the publication. In keyword co-occurrence analysis, a total of 410 nodes and 1744 links were detected. Each node represents a keyword; the frequency of keyword occurrence is indicated by the size of the node, and the connection between keywords represents the link in the dataset.^[32]^ Figure 7 shows the network of keyword co-occurrence analysis. Most of the most frequent keywords are close to each other, indicating the main research in the field of TME-IE. As mentioned above, modularization (*Q*) is an important index revealing that the network is divided into clustering intensities. The higher the value is, the greater the network partition.^[33,34]^ The *Q* value in this study is 0.7486, which exceeds the threshold of 0.3, indicating that the keyword co-occurrence network contains loosely coupled clustering. This finding supports the view that the research topics and concerns related to TME-IE are relatively scattered.

The size of the nodes in the keyword co-occurrence network specifies the frequency of keyword occurrence. The keywords calculated by frequency are “expression,” “tumor microenvironment,” “cancer,” “cells” and “T cells.” The keywords “colony stimulating factor,” “blockade,” “lung cancer,” “apoptosis,” and “suppression” are the most influential keywords in terms of centrality (Table 6). These keyword lists identify the main hotspots in the field of TME-IE. Centrality measures the extent to which a particular node is on the shortest path between other nodes. The higher the centrality value is, the stronger the influence of specific nodes. In CiteSpace, the threshold of high centrality is approximately 0.1. Keywords with high centrality connect multiple research topics and play a central role in the development of TME-IE research.

**Table 6:** Effective keywords in the field of TME-IE research, ranked by frequency (>115) and centrality (>0.08).

Keywords	Frequency	Yr	Keywords	Centrality	Yr
Expression	783	2004	Colony stimulating factor	0.23	2005
Tumor microenvironment	581	2006	Blockade	0.17	2005
Cancer	569	2006	Lung cancer	0.15	2005
Cells	337	2006	Apoptosis	0.14	2008
t cells	309	2005	Suppression	0.12	2007
Activation	268	2005	Colorectal cancer	0.11	2005
Immunotherapy	260	2007	Lymphocytes	0.11	2006
Immune escape	243	2005	Antigen presentation	0.11	2006
Immune evasion	243	2004	Tumor immunology	0.11	2007
Microenvironment	204	2009	Squamous cell carcinoma	0.11	2007
Survival	189	2009	Tumor immunity	0.11	2005
Blockade	189	2005	Fas ligand	0.11	2004
Resistance	166	2012	Tumor microenvironment	0.09	2006
Breast cancer	162	2004	Breast cancer	0.09	2004
Progression	162	2009	In vivo	0.09	2006
Regulatory t cells	139	2007	Cytokine	0.09	2004
Mechanisms	125	2007	Antigen-presenting cells	0.09	2006
Growth	118	2012	Responses	0.08	2007
Tumor	117	2007	Down regulation	0.08	2006
Dendritic cells	115	2004	Gene therapy	0.08	2007

#### 3.6.2. Keyword cluster analysis

**Figure 8.**
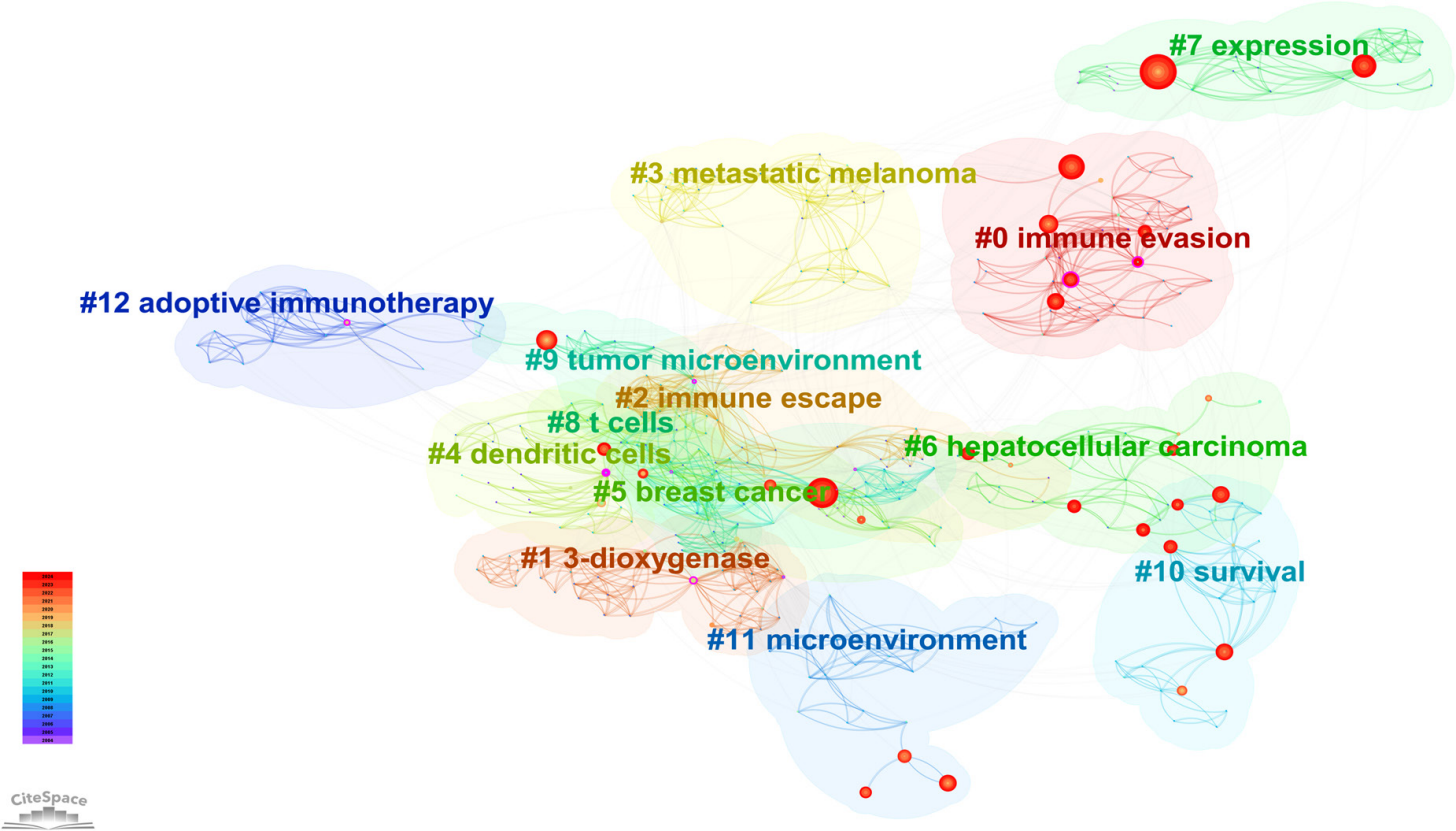
Cooccurring keyword-based knowledge clusters. (Modularity *Q* = 0.7486, *S* = 0.9005).

In cluster analysis, bibliometric information is collected and different subgroups are derived to present the structure of the research topic. On the basis of their co-occurrence, CiteSpace estimates the relationships between keywords, and then classifies these keywords into different clusters.^[35,36]^ CiteSpace has an automatic tagging function, which is used to describe derived clustering. In particular, it extracts terms from keywords in each cluster and uses them as tags. Clusters are numbered in descending order of size, starting with the largest # 0, the second largest # 1, and so on. As shown in Figure 8 and Table 7, TME-IE research top 13 clusters are listed as: # 0 IE, # 13-dioxygenase, # 2 immune escape, # 3 metastatic melanoma, # 4 dendritic cells, # 5 breast cancer, # 6 hepatocellular carcinoma, # 7 expression, # 8 t cells, # 9 tumor microenvironment, # 10 survival, # 11 microenvironment, and # 12 adoptive immunotherapy. Some clusters have the same topic, so they are merged and interpreted.

**Table 7:** Knowledge clusters based on keyword co-occurrence.

Rank	Size	Mean contour value	Peak yr	Top 5 high-frequency keywords (The first keyword serves as the label for each cluster)
0	39	0.869	2007	Immune evasion (81.49, 1.0E-4); activation (32.38, 1.0E-4); cancer (31.65, 1.0E-4); pathway (16.77, 1.0E-4); immune checkpoint (10.93, 0.001)
1	37	0.932	2008	3-dioxygenase (16.75, 1.0E-4); lung cancer (16.35, 1.0E-4); proliferation (12.54, 0.001); indoleamine 2 (11.82, 0.001); squamous cell carcinoma (9.22, 0.005)
2	35	0.869	2007	Immune escape (111.47, 1.0E-4); immune evasion (20.73, 1.0E-4); tils (11.45, 0.001); prognosis (8.58, 0.005); tumor microenvironment (8.54, 0.005)
3	32	0.862	2007	Metastatic melanoma (12.29, 0.001); melanoma patients (11.87, 0.001); tumor microenvironment (9.56, 0.005); lymphocytes t (8.67, 0.005); molecules (8.67, 0.005)
4	32	0.868	2007	Dendritic cells (16.67, 1.0E-4); immune evasion (12.83, 0.001); colony stimulating factor (11.9, 0.001); tumor microenvironment (11.31, 0.001); dendritic cell (9.78, 0.005)
5	29	0.891	2007	Breast cancer (57.78, 1.0E-4); tumor immune evasion (15.89, 1.0E-4); therapy (15.39, 1.0E-4); hepatocellular carcinoma (11.74, 0.001); doxorubicin (10.59, 0.005)
6	27	0.819	2008	Hepatocellular carcinoma (48.07, 1.0E-4); progression (18.37, 1.0E-4); growth (18.37, 1.0E-4); resistance (17.97, 1.0E-4); t cells (17.56, 1.0E-4)
7	27	0.952	2006	Expression (43.39, 1.0E-4); cells (35.34, 1.0E-4); immune evation (15.32, 1.0E-4); cd73 (12.11, 0.001); dendritic cells (11.55, 0.001)
8	24	0.966	2007	t cells (52.38, 1.0E-4); carcinoma (17.83, 1.0E-4); t cell (11.81, 0.001); prognosis (11.02, 0.001); t cell activation (10.48, 0.005)
9	24	0.944	2006	Tumor microenvironment (173.92, 1.0E-4); microenvironment (17.31, 1.0E-4); tumor-infiltrating (14.15, 0.001); tumor microenvironment (tme) (9.85, 0.005); t-lymphocytes (9.43, 0.005)
10	23	0.932	2008	Survival (28.85, 1.0E-4); metastasis (17.46, 1.0E-4); regulatory t cells (15.67, 1.0E-4); treg (10.07, 0.005); system (9.58, 0.005)
11	22	0.904	2008	Microenvironment (33.58, 1.0E-4); mechanisms (28.31, 1.0E-4); antigen (11.41, 0.001); cutting edge (9.3, 0.005); interleukin 17 (9.3, 0.005)
12	19	0.923	2009	Adoptive immunotherapy (18.37, 1.0E-4); lymphocytes (13.04, 0.001); patient survival (9.17, 0.005); penile cancer (9.17, 0.005); blood gene expression profiling (9.17, 0.005)

#### 3.6.3. Emerging trends analysis

Keyword emergence detection is an effective method for detecting keywords that have attracted great attention in the research field at different stages of development, and it is more advanced than citation counting. The citation count is the first indicator of intelligence influence, whereas the citation burst is an advanced index used to guide the attention of the research field. Therefore, in the development stage of this field, having a high number of sudden keywords is a milestone in discovering the frontier of research in this field. The keywords with the highest reference bursts and their burst intensities and durations are summarized in Table 8. The keywords “dendritic cells” and “antigens” appeared for the first time in 2004, they began to emerge in 2004 and ended in 2016 and 2015, respectively. “Inflammation” was the first popular keyword between 2010 and 2014, with a pop intensity of 11.08. Similarly, “suppressor cells” is another keyword with a sudden strong quotation at this stage, and its emergent intensity is 20.26. In addition, “lung cancer,” “PD-1” and “inhibition” were also detected via burst analysis. Finally, “resistance” and “hepatocellular carcinoma” are recent outbursts, representing emerging trends and noteworthy frontiers in the field of TME-IE.

**Table 8:** Top 25 keywords with the strongest citation bursts.

Keywords	Yr	Strength	Begin	End	2004–2024
Dendritic cells	2004	28.92	2004	2016	▃▃▃▃▃▃▃▃▃▃▃▃▃▂▂▂▂▂▂▂▂
Antigen	2004	8.71	2004	2015	▃▃▃▃▃▃▃▃▃▃▃▃▂▂▂▂▂▂▂▂▂
Receptor	2005	8.21	2005	2016	▂▃▃▃▃▃▃▃▃▃▃▃▃▂▂▂▂▂▂▂▂
Lymphocytes	2006	13.4	2006	2018	▂▂▃▃▃▃▃▃▃▃▃▃▃▃▃▂▂▂▂▂▂
Peripheral blood	2006	8.86	2006	2014	▂▂▃▃▃▃▃▃▃▃▃▂▂▂▂▂▂▂▂▂▂
In vivo	2006	7.04	2006	2011	▂▂▃▃▃▃▃▃▂▂▂▂▂▂▂▂▂▂▂▂▂
Tolerance	2007	5.84	2007	2014	▂▂▂▃▃▃▃▃▃▃▃▂▂▂▂▂▂▂▂▂▂
Carcinoma	2007	23.09	2008	2018	▂▂▂▂▃▃▃▃▃▃▃▃▃▃▃▂▂▂▂▂▂
Immune response	2008	5.87	2008	2016	▂▂▂▂▃▃▃▃▃▃▃▃▃▂▂▂▂▂▂▂▂
Inflammation	2004	11.08	2010	2017	▂▂▂▂▂▂▃▃▃▃▃▃▃▃▂▂▂▂▂▂▂
Induction	2010	5.88	2010	2014	▂▂▂▂▂▂▃▃▃▃▃▂▂▂▂▂▂▂▂▂▂
Suppressor cells	2011	20.26	2011	2017	▂▂▂▂▂▂▂▃▃▃▃▃▃▃▂▂▂▂▂▂▂
Angiogenesis	2009	5.77	2013	2014	▂▂▂▂▂▂▂▂▂▃▃▂▂▂▂▂▂▂▂▂▂
Lung cancer	2005	13.65	2014	2018	▂▂▂▂▂▂▂▂▂▂▃▃▃▃▃▂▂▂▂▂▂
t cells	2005	7.92	2014	2016	▂▂▂▂▂▂▂▂▂▂▃▃▃▂▂▂▂▂▂▂▂
Melanoma	2007	6.01	2014	2017	▂▂▂▂▂▂▂▂▂▂▃▃▃▃▂▂▂▂▂▂▂
Breast cancer	2004	5.65	2015	2018	▂▂▂▂▂▂▂▂▂▂▂▃▃▃▃▂▂▂▂▂▂
PD-1	2007	11.42	2018	2019	▂▂▂▂▂▂▂▂▂▂▂▂▂▂▃▃▂▂▂▂▂
Responses	2007	8.87	2018	2020	▂▂▂▂▂▂▂▂▂▂▂▂▂▂▃▃▃▂▂▂▂
Inhibition	2008	8.85	2019	2020	▂▂▂▂▂▂▂▂▂▂▂▂▂▂▂▃▃▂▂▂▂
Resistance	2012	14.59	2021	2024	▂▂▂▂▂▂▂▂▂▂▂▂▂▂▂▂▂▃▃▃▃
Therapy	2007	10.88	2021	2022	▂▂▂▂▂▂▂▂▂▂▂▂▂▂▂▂▂▃▃▂▂
Mechanisms	2007	7.83	2021	2022	▂▂▂▂▂▂▂▂▂▂▂▂▂▂▂▂▂▃▃▂▂
Hepatocellular carcinoma	2007	16.17	2022	2024	▂▂▂▂▂▂▂▂▂▂▂▂▂▂▂▂▂▂▃▃▃
Tumor	2007	9.11	2022	2024	▂▂▂▂▂▂▂▂▂▂▂▂▂▂▂▂▂▂▃▃▃

PD-1 = programmed cell death protein 1.

Keywords represent the thematic content of publications. Therefore, keyword clustering and burst analysis help identify research hotspots and reveal the evolutionary trajectory of the field. As illustrated in Figure 7 and Table 7, we identified 13 major clusters using CiteSpace. Further consolidation of these clusters revealed that they primarily encompass mechanistic studies, therapeutic strategies, and tumor-specific investigations in immune escape research, with immune escape mechanisms remaining a consistently prominent topic. Table 8 (Keyword burst detection) further analyzes keywords that garnered significant attention during different developmental phases. Since 2004, key terms related to fundamental mechanisms of tumor-associated IE: such as dendritic cells, antigen, receptor, lymphocyte, and peripheral blood: have exhibited sustained prominence, reflecting research spanning cellular, molecular, and systemic levels. Keywords like lung cancer, melanoma, and breast cancer emerged as burst terms, indicating a shift toward tumor-specific analyses. Researchers aim to uncover commonalities and distinctions in immune-related features across malignancies, using individual tumor types as entry points for broader insights. With deeper mechanistic understanding, a growing number of studies have pivoted to immunotherapy, establishing it as a major research hotspot in recent years: and likely a future priority.

## 4. Discussion

### 4.1. General information

In this study, we analyzed a total of 2727 articles published in 654 academic journals, with contributions from 2974 institutions across 87 countries/regions. Since 2004, both the annual publication count and citation frequency have demonstrated consistent growth, transitioning from gradual increases in early years to rapid acceleration in recent periods, indicating this field has become a focal point of global research interest. The top 4 journals by publication volume were *Frontiers in Immunology*, *Cancer Research*, *Frontiers in Oncology*, and *Journal for Immunotherapy of Cancer*, with *Cancer Research* ranking first in co-citation frequency, underscoring its dominant influence in this domain. The journal co-citation network revealed that research in this field spans multiple disciplines, including cancer biology, immunology, molecular biology and genetics, clinical medicine, and translational research, reflecting the interdisciplinary nature of TME-IE studies. This interdisciplinary structure suggests that future breakthroughs may emerge at the intersections of these fields, and researchers should consider collaborations across traditional disciplinary boundaries. The co-occurrence network visualization of subject categories further confirmed the breadth, diversity, and complexity of TME-IE research, with oncology, immunology, and cell biology emerging as the core categories. Notably, categories such as “medical research and experimental” and “biotechnology and applied microbiology” exhibited high betweenness centrality, indicating their critical bridging role in connecting different research areas. This finding suggests that future interdisciplinary collaborations involving these fields may yield novel insights into TME-IE mechanisms.

Bibliometric analysis of authors, institutions, and nations identified China, the United States, Germany, Italy, Japan, and the UK as major contributors (each with >100 publications), with China and the US collectively accounting for over 70% of total output, establishing clear dominance. All ten most productive institutions originated from these 2 nations, exhibiting robust collaborative networks. The most prolific author, Wei Wang, emerged as a central node in co-authorship analysis. While leading institutions and authors formed extensive collaborative clusters, most researchers published either independently or in small groups. This pattern highlights the importance of fostering international and inter-institutional collaborations to accelerate progress in TME-IE research. For early-career researchers, identifying and connecting with these key collaborative networks could provide valuable opportunities for mentorship and resource sharing. Moreover, the dominant position of China and the US likely reflects substantial governmental research investments in biomedical sciences over the past 2 decades ^[37]^, suggesting that sustained funding is crucial for maintaining research competitiveness in this rapidly evolving field.

### 4.2. Research hotspots

Through comprehensive analysis of keyword co-occurrence, frequency distribution, clustering, timeline visualization, and burst detection, we have delineated the research hotspots in the TME-IE field, which are primarily concentrated on mechanistic studies and clinical therapeutics. Integrating these findings with the existing literature, we have mapped the intellectual landscape of this field.

#### 4.2.1. Mechanistic studies of IE

Tumor immune escape is one of the important mechanisms for the occurrence and development of malignant tumors. A variety of intrinsic and extrinsic factors can significantly regulate the function of immune cells and drive immune escape.^[38]^ Intrinsic factors mainly refer to the intrinsic functions and phenotypic changes of cancer cells, including but not limited to chromosomal and epigenetic changes,^[39,40]^ differential gene expression, and changes in the secretome and metabolome.^[41,42]^ These factors are often not clearly distinguished. From the age, race, gender and hormone regulation of individual patients,^[43–45]^ to the daily lifestyle of patients,^[46]^ to the extracellular environment of tumors, these can be classified as external factors, which are closely linked to intrinsic factors and jointly affect the immune escape of cancer cells.^[38]^ In recent years, with the rapid development of disciplines such as immunology, molecular biology and genomics, people understanding of the mechanism of tumor immune escape based on the tumor microenvironment has continued to deepen. The 3E theory^[2]^ plays a pivotal role in explaining TME-IE. In the initial stage of malignant transformation of cells, the immune system begins to identify and destroy malignant cells and perform “Elimination” operations.^[2]^ If the elimination process is completed, the subject will be free from cancer and the entire cascade will end. However, in a few cases, some malignant cells can enter a functional dormant state. Although the immune system is still trying to eliminate most of the malignant cells, it cannot accurately identify and destroy these few cells, so it enters the “Equilibrium” stage.^[2,3]^ Finally, as more and more malignant cells escape the immune system, the body clinically manifests as a disease state. This final stage can be summarized as “immune escape.”^[2,4]^ With the deepening of research, some scientists have now proposed a new 3C theory^[47]^ to further summarize the mechanism of tumor immune escape.

Since the tumor microenvironment is a complex and dynamic ecosystem, the specific mechanism of tumor immune escape also involves multiple aspects. First, tumor cells evade the recognition of the immune system by changing the expression of antigens. This includes the loss or downregulation of tumor antigens^[48]^ and the abnormal expression of major histocompatibility complex molecules.^[49]^ These changes make it difficult for tumor cells to be recognized by T cells, thereby escaping immune surveillance. In addition, tumor cells can also interfere with the recognition function of immune cells by releasing soluble antigens or exosomes.^[50,51]^ Second, tumor cells can actively create an immunosuppressive microenvironment.^[52]^ There are a large number of immunosuppressive cells in the tumor microenvironment, such as regulatory T cells, MDSC and tumor-associated macrophages.^[53]^ These cells inhibit the function of effector T cells and promote tumor growth by secreting inhibitory cytokines (such as transforming growth factor-beta, Interleukin-10, etc) and expressing inhibitory molecules.^[54–56]^ Recent advances have further highlighted the complex interactions between tumor cells, immune cells, and stromal cells mediated by multiple cytokines and chemokines in the tumor microenvironment, particularly in aggressive subtypes such as triple-negative breast cancer.^[57]^ These interactions play a critical role in IE and response to immunotherapy, underscoring the importance of understanding tumor-stroma cross-talk in specific cancer contexts. At the same time, there are adverse conditions such as hypoxia, acidity and nutrient deficiency in the tumor microenvironment, which further inhibit the function of immune cells.^[58–60]^ Abnormal expression of immune checkpoint molecules is another important mechanism of tumor immune escape. Tumor cells and immunosuppressive cells highly express immune checkpoint molecules such as PD-L1 and CTLA-4, which inhibit the activation and function of T cells by binding to the corresponding receptors on the surface of T cells.^[61]^ This is an important strategy for tumors to evade immune attack. In addition, tumor cells can also evade the phagocytosis of immune cells by upregulating signaling molecules such as CD47.^[62]^ Finally, the metabolic reprogramming of tumor cells also plays an important role in immune escape.^[63]^ Tumor cells not only meet their own rapid proliferation needs by changing the glucose, amino acid and lipid metabolism pathways, but also affect the function of immune cells. For example, tumor cells take up a large amount of glucose and release lactate, causing glucose deficiency and increased acidity in the microenvironment, thereby inhibiting the activity of T cells and natural killer cells.^[63,64]^ In addition, tumor cells can also inhibit the proliferation and function of T cells by consuming amino acids such as methionine and serine.^[65,66]^

#### 4.2.2. Clinical therapeutics

In response to the complex mechanisms of tumor immune escape, researchers have developed a variety of treatment strategies. Immune checkpoint inhibitors are one of the most successful tumor immunotherapy methods. PD-1/PD-L1 inhibitors and cytotoxic T-lymphocyte-associated protein 4 inhibitors have achieved remarkable results in the treatment of various tumors by blocking the immune checkpoint pathway and restoring the antitumor activity of T cells.^[67]^ However, the efficacy of a single immune checkpoint inhibitor is limited. The combination of different immune checkpoint inhibitors or other treatments may improve the antitumor efficacy.^[68]^ Tumor vaccines^[69]^ are another important immunotherapy strategy. By introducing tumor antigens, antigen-presenting cells or genes encoding antigens into the body, specific antitumor immune responses can be activated. Currently, a variety of tumor vaccines are undergoing clinical trials, including peptide vaccines, dendritic cell vaccines and nucleic acid vaccines.^[69]^ Personalized tumor vaccines are designed based on the patient tumor-specific antigens and are expected to improve the treatment effect. Adoptive cell therapy is a method of killing tumor cells by amplifying and modifying immune cells in vitro and then returning them to the patient body.^[5]^ Chimeric antigen receptor T cell (CAR-T) therapy has made breakthrough progress in the treatment of hematological tumors.^[5]^ In addition, tumor-infiltrating lymphocyte therapy and T cell receptor engineered T cell therapy have also shown potential in the treatment of solid tumors.^[70]^ These methods overcome tumor immune escape by enhancing the tumor recognition ability and killing activity of T cells. Combination therapy strategy is an important direction to improve the effect of tumor immunotherapy. The combined use of immunotherapy with traditional methods such as chemotherapy, radiotherapy, and targeted therapy can produce synergistic effects. For example, chemotherapy drugs can induce immunogenic cell death and increase tumor antigen release; radiotherapy can change the tumor microenvironment and enhance immune cell infiltration. In addition, the combined use of inhibitors targeting tumor metabolic reprogramming and immunotherapy has also shown good prospects. These combined strategies are expected to overcome the limitations of single treatments and improve the overall therapeutic effect.

### 4.3. Future projections and practical implications

Combining our bibliometric findings with the mechanistic and therapeutic discussions above, we propose several future directions and practical implications in the TME-IE field.

First, for researchers planning to publish in this field, the journal analysis provides practical guidance. High-impact journals such as *Cancer Research*, *Nature*, and *Cell* dominate the co-citation network, indicating their role in shaping the intellectual foundation of TME-IE research. However, specialized journals like *Frontiers in Immunology* and *Journal for Immunotherapy of Cancer* publish the highest volume of original research, making them attractive targets for researchers seeking appropriate venues for their work. Understanding this publication landscape can help authors strategically select journals that align with their research scope and career goals.

Second, the collaborative network analysis revealed that while a few core authors and institutions form dense collaborative clusters, the majority of researchers work in relative isolation. This finding has important implications: researchers seeking to maximize their impact should actively pursue collaborations with central players in the field, as evidenced by the high centrality scores of institutions like the University of Texas System and the Chinese Academy of Sciences. Additionally, the dominance of China and the US in publication output suggests that researchers from other countries may benefit from establishing partnerships with institutions in these nations.

Third, the high centrality of interdisciplinary categories such as “biotechnology and applied microbiology” and “engineering, biomedical” suggests that future breakthroughs may emerge at the intersection of traditional immunology/oncology with emerging technologies. Researchers should consider collaborations with experts in nanotechnology, bioengineering, and computational biology to develop novel therapeutic platforms or predictive models.

Finally, the temporal evolution of keyword bursts indicates that while immune checkpoint inhibitors have been extensively studied, current research efforts are shifting toward understanding and overcoming resistance mechanisms, as well as expanding immunotherapy to cancers traditionally considered “cold” or immunotherapy-resistant. For early-career researchers, focusing on resistance mechanisms or immunotherapy combinations in specific challenging cancer types may offer promising research directions with high-impact potential.

In summary, this bibliometric analysis not only maps the historical development and current state of TME-IE research but also provides actionable insights for researchers at all career stages. By identifying key players, emerging trends, and interdisciplinary opportunities, our findings can guide future research directions, foster collaborations, and ultimately accelerate progress toward more effective cancer immunotherapies.

## 5. Limitations

It is important to acknowledge that bibliometrics, by summarizing publication frequency, reflects research attention and activity rather than serving as a direct measure of the biological or clinical significance of a given phenomenon. A highly studied topic may reflect technical feasibility, funding priorities, or historical momentum, rather than its intrinsic importance in tumor biology. Conversely, mechanisms that are difficult to study technically may be underrepresented in the literature despite their potential biological significance. Furthermore, bibliometric analyses tend to focus on English-language literature, potentially overlooking valuable contributions published in other languages. Finally, due to the complexity and time required for collecting and analyzing citation data, our bibliometric approach may not capture the entirety of the literature in this field or the most recent developments.

## 6. Conclusion

This study presents a comprehensive bibliometric analysis of global research trends in the TME-IE over the past 2 decades. The field has experienced exponential growth, with China and the United States leading in productivity and collaboration. Keyword analysis reveals a clear evolution from fundamental mechanistic studies toward clinically oriented applications, including immunotherapy resistance and combination strategies. Beyond mapping the intellectual landscape, our findings offer practical guidance for researchers at all career stages: helping early-career investigators identify emerging hotspots and productive collaborations, while highlighting interdisciplinary opportunities at the intersection of immunology, bioengineering, and computational biology. Collectively, this work provides actionable insights to guide future investigations and accelerate the development of more effective cancer immunotherapies.

## Author contributions

**Validation:** Han Bi.

**Visualization:** Han Bi, Meng Gao, Yuying Ren.

**Investigation:** Qifu Bo.

**Data curation:** Ao Li, Meng Gao, Lingling Wang.

**Software:** Jinrong Liu, Xiaoyu Zhao.

**Formal analysis:** Lingling Wang.

**Writing – original draft:** Xinyu Feng, Xiumei Sun, Yiyi Ding, Yuying Ren, Xiaoyu Zhao.

**Writing – review & editing:** Qifu Bo, Wenhao Wang.






